# Multispecies transcriptome analysis identifies networks protecting against MASLD in Göttingen minipigs

**DOI:** 10.1016/j.jlr.2026.101089

**Published:** 2026-06-25

**Authors:** Pascal Gottmann, Wenke Jonas, Simone Renner, Julia Philippou-Massier, Theresa Hommel, Maik Dahlhoff, Anja Zeigerer, Eckhard Wolf, Heike Vogel, Annette Schürmann

**Affiliations:** 1 Department of Experimental Diabetology, German Institute of Human Nutrition Potsdam-Rehbruecke (DIfE), Nuthetal, Germany; 2 German Center for Diabetes Research (DZD), Neuherberg, Germany; 3 Chair for Molecular Animal Breeding and Biotechnology, Gene Center and Department of Veterinary Sciences, LMU Munich, Munich, Germany; 4 Laboratory for Functional Genome Analysis (LAFUGA), Gene Center, LMU Munich, Munich, Germany; 5 Laboratory Animal Medicine, Department of Biological Sciences and Pathobiology, University of Veterinary Medicine Vienna, Vienna, Austria; 6 Basic Principles of Metabolic Diseases, Medical Faculty Mannheim, Mannheim, Germany; 7 University of Potsdam, Institute of Nutritional Sciences, Nuthetal, Germany

**Keywords:** liver, cross-species comparison, lipoproteins, apolipoproteins, MASLD, pig breeds, AMPK signaling

## Abstract

To understand the mechanisms underlying the remarkable resistance of Göttingen minipigs to metabolic dysfunction-associated steatotic liver disease (MASLD), a multilevel transcriptomic analysis comparing their liver with those of humans, mice, and MASLD susceptible pig breeds was performed. This analysis revealed 692 genes uniquely differentially expressed in livers of Göttingen minipigs, linked to multiple metabolic and signaling pathways (e.g., AMPK signaling). Among these, 11 transcription factors (TFs), 3 hepatokines (*DPP4*, *ITIH1*, and *ITIH3*), and 6 additional secreted proteins (including *PCSK9* and *APOM*) exhibited particularly strong differential expression and emerged as candidate mediators of MASLD resistance. This finding was further supported by weighted gene coexpression network analysis, which identified a core regulatory network driven by the TFs HMBOX1 and PATZ1, presumably regulating 41% of its differentially expressed genes. Collectively, these results suggest that a distinct transcriptional program involving HMBOX1/PATZ1 and their downstream targets, including secreted factors, contributes to the MASLD-resistant phenotype of Göttingen minipigs and may provide promising targets for the prevention and treatment of fatty liver disease.

Metabolic dysfunction-associated steatotic liver disease (MASLD), previously known as non-alcoholic fatty liver disease, represents a growing global health concern characterized by fat accumulation in the liver and is frequently linked to metabolic dysfunction, type 2 diabetes, and an increased risk of fibrosis and cirrhosis (1). Understanding the mechanisms underlying susceptibility and resistance to MASLD is crucial for developing targeted therapies and preventive strategies. Early research utilized murine models to investigate the molecular basis of MASLD susceptibility. However, these models often only partially recapitulate the human disease phenotype, reflecting significant differences in MASLD development and transcriptional changes in the liver of mice and humans (2, 3). To address this, we have previously explored an alternative model system of human hepatocytes within three-dimensional collagen matrices (4). Beyond this and similar approaches (5), the emergence of specific pig breeds with varying susceptibility to MASLD offers another promising avenue, potentially bridging the gap between the homogeneity of inbred mouse strains and the heterogeneity of human populations. This enables genome-wide association studies to link genetic variation to observable phenotypes (6). Intriguingly, Göttingen minipigs exhibit remarkable resistance to MASLD, demonstrating significantly less fat accumulation and fibrosis compared to other pig breeds and human populations (7, 8, 9). Comparative analysis between Göttingen minipigs and species prone to develop fatty liver provide therefore a unique opportunity to uncover mechanisms that protect against MASLD in a broader context.

To investigate these mechanisms, we performed a multilayered transcriptomic analysis of hepatic responses in Göttingen minipigs and mice fed high-fat diets, as well as in human hepatocytes treated with FAs. In addition, transcriptomic datasets from MASLD-susceptible miniature pig breeds, including Tibetan and Wuzhishan pigs, were also incorporated to enable cross-species and cross-breed comparisons of disease-associated pathways and protective molecular signatures.

## Materials and methods

### Preprocessing of sequencing data

Raw liver RNA-seq datasets were obtained from NCBI Gene Expression Omnibus (GEO) for Tibetian/Wuzhishan pigs (GSE142346) and for mice (GSE88818). Liver RNA-seq data of Göttingen minipigs fed a high-fat diet for a period of 70 weeks (8) were generated by the Laboratory for Functional Genome Analysis. Reads were aligned to the *Sus scrofa* Sscrofa11.1 genome for pig species and to the *Mus musculus* GRCm38.p6 genome for mouse using STAR (10). In addition, RNA-seq reads of Göttingen minipig were aligned to a specific minipig genome annotation (Wuzhishan); as this approach provided limited additional information, it was not applied to the other pig species. Transcript assembly and quantification of transcript abundance were performed using StringTie2 (11) using default parameters yielding FPKM (Fragments Per Kilobase of transcript per Million mapped reads) values.

### Diets of pigs

Göttingen minipigs were fed a diet providing 35% calories from fat, 44% calories from carbohydrates, and 21% calories from protein (8). The Tibetian and Wuzhishan pigs were fed a similar diet providing 31% calories from fat, 50% calories from carbohydrates, and 19% calories from protein with a supplementation of cholesterol (12).

### Estimation of differentially expressed genes (DEGs) in livers of pigs and mice

For the identification of significantly differentially expressed genes (DEGs) in the liver, Student’s *t* test with Welch’s correction was performed. A significance threshold of *P* < 0.05 was applied to define DEGs. Furthermore, Göttingen minipig data were analyzed using DeSeq2 with correction for multiple testing via FDR.

### Sequencing data of human primary hepatocytes

Data from NCBI GEO dataset: GSE247407, containing precalculated FPKM-values, were utilized. DEGs were identified as described by Kwon *et al.* (4) using the limma package (version 3.54) in R, with correction for multiple testing via the Benjamini-Hochberg method.

### Analysis of ChIP-seq data

ChIP-seq data for histone modifications H3K4me3, H3K4me1, and H3K27ac in pig livers were downloaded from the European Nucleotide Archive under the accession PRJEB14330. Reads were aligned to the *Sus scrofa* Sscrofa11.1 genome using STAR (10). Peak calling was performed using MACS2 (13), following the ENCODE best practices (14). Transcription factor (TF)-specific ChIP-seq for PATZ1 (GSM5215711) were obtained via the ChIP-Atlas platform and analyzed using the integrated interface to the IGV genome browser.

### Target prediction for differentially expressed TFs

To identify promoter regions of DEGs, the 2 kb pairs region upstream of the transcription start site was screened for the presence of the histone marks H3K4me3 and H3K27ac. Similarly, enhancers were defined as regions 50 kb upstream of the transcription start site (excluding the proximal 2-kb promoter region) that intersect histone marks H3K4me1 and H3K27ac. The identified regions were screened for TF binding motifs received from the JASPAR database (15) using TFBSTools (16) with default settings. Finally, we correlated the expression of TFs with the expression of their target genes to confirm potential targets.

### Weighted gene coexpression network analysis (WGCNA) of Göttingen minipig RNA-seq data

Weighted gene coexpression network analysis (WGCNA) was performed on the Göttingen minipig RNA-seq data following the guidelines provided by Horvath *et al.* (17). To ensure scale-free topology, a power value of 10 was selected. Network construction parameters included network type unsigned, a minimal module size of 30, and a clustering linkage cut height of 0.25.

### Pathway enrichment analysis

Pathway enrichment was conducted using DAVID tools knowledge base v2025_v1 (18). As full annotations for the pig genome in regards to signaling pathways are missing, we decided to use the full mouse genome universe as background gene list.

### PigTex genome-wide association study (GWAS) data

Genome-wide association study (GWAS) data of pigs were obtained from the PigBiobank resource (6). Candidate genes were identified by screening selected traits for associations with a significance threshold of < 0.005.

### Bioinformatics analysis and plotting

Analyses were performed in R-Studio using the R-programming language (version 4.4.3), including computing correlations, identifying intersection of sets, and generating plots. Plots were produced with ggplot2 (version 3.5.1). Networks visualizations were created with the igraph R-package (version 2.0.3).

### Creation of pig-mouse-human gene mapping

To harmonize gene identifiers across the three species, we used the biomaRt R-package to map pig and human Ensembl gene IDs to their mouse orthologs, allowing one-to-many and many-to-many relationships. Later on, analysis focusing on cross-species comparison was done on basis of mouse orthologs, since the mouse genome has the strongest history on experimental setups.

### qRT-PCR analyses of pig liver

Total RNA was purified from snap-frozen porcine left liver lobe and right liver lobe according to RNAseq data using TRIzol™ extraction (Thermo Fisher Scientific, Waltham, MA). Reverse transcription of 2,500 ng total RNA per sample was performed using the SuperScript™ IV First-Strand Synthesis System (Thermo Fisher Scientific, Waltham, MA) according to the manufacturer’s instructions. cDNA derived from left liver lobe and right liver lobe of each pig was pooled equally. Primers for target genes were designed using the NCBI Primer-BLAST tool (Supplemental Table S1) (19). Reference gene primers (*ACTB* and *B2M*) were obtained from Wang *et al.* (20). SYBR® Green I-based RT-qPCR was performed using Luna® Universal qPCR Master Mix (New England Biolabs, MA) on a qTower384 real-time PCR system (Analytik Jena, Jena, Germany). Each reaction contained 1 μl of diluted cDNA (1:5) and primers at a final concentration of 150 nM respectively, in a total volume of 10 μl. Reactions were set up in triplicate using an epMotion 5075 automated pipetting system (Eppendorf, Vienna, Austria). Each run included no-template controls and no-RT controls. Thermal cycling was performed using a three-step protocol with an annealing temperature of 60°C. Specificity of amplification was confirmed by melt curve analysis (60–95°C, 0.3°C/s). *ACTB* and *B2M* were used as reference genes. Relative gene expression was calculated using the 2-ΔΔCT method (21).

## Results

### Multilevel approach to identify genes and pathways protecting Göttingen minipig from MASLD

**Fig. 1:**
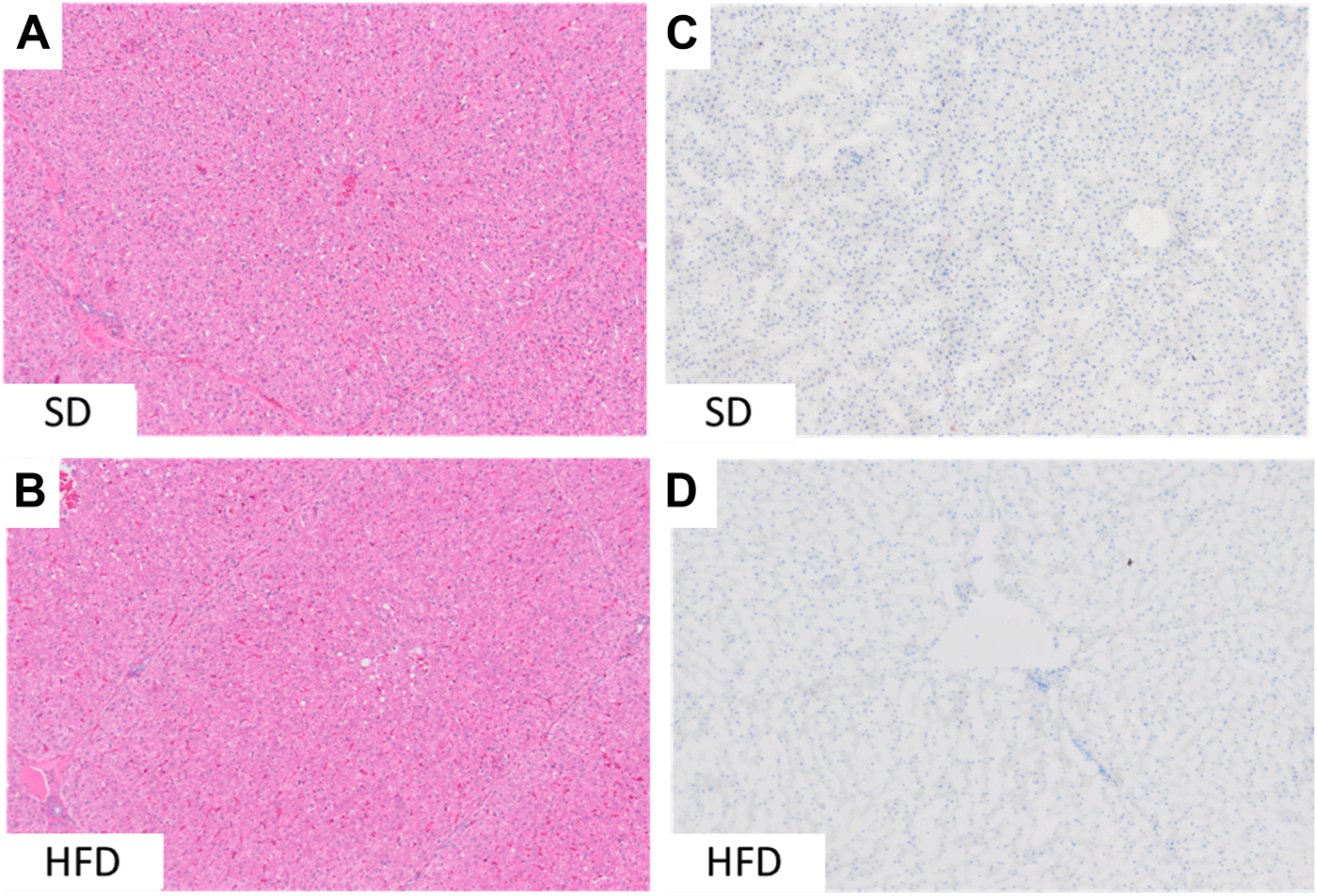
Liver histology of Göttingen minipig after 70 weeks of high-fat diet feeding. A and B: Representative microscopy pictures of H&E staining. C and D: Representative microscopy pictures of Oil Red O staining. (A, B, C, and D: 100 x magnification; HFD: high-fat diet; SD: standard diet).

**Fig. 2:**
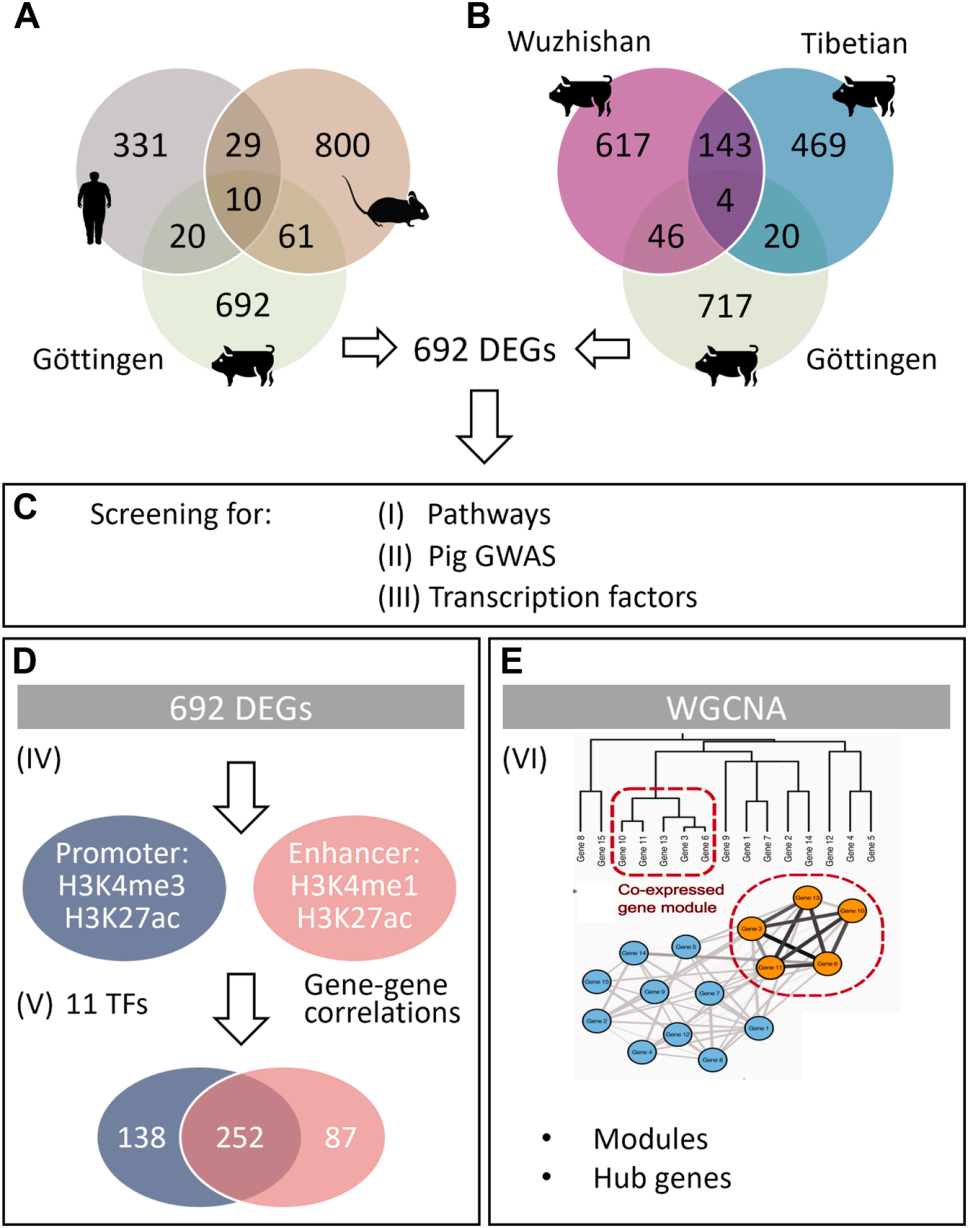
Schematic workflow of the transcriptomic and bioinformatic analyses to identify genes that protect against MASLD. A: Venn diagram depicting differentially expressed genes (DEGs) across livers of mice, Göttingen minipigs, and primary human hepatocytes. B: Venn diagram of DEGs across livers from Göttingen, Wuzhishan, and Tibetian minipigs fed high-fat diets. C: Screening for disease-associated pathways, overlap with pig GWAS candidate genes, and regulatory transcription factors. D: Transcription factor (TF) target prediction was performed by integrating (IV) chromatin features - promoters (H3K4me3), enhancers (H3K4me1), and activity (H3K27ac) - and (V) TF binding motif analysis for 11 uniquely differentially expressed in Göttingen minipigs after feeding a high-fat diet. E: Identification of coexpression modules and screening for TFs and hub genes. MASLD, metabolic dysfunction-associated steatotic liver disease.

Göttingen minipigs exhibit resistance to metabolic MASLD even under high-fat diet, as indicated by preserved tissue architecture (Fig. 1A, B) and no increase in lipid accumulation assessed by Oil Red O staining (Fig. 1C, D). To investigate the mechanisms contributing to this protection, a comprehensive, multilevel transcriptomic analysis was conducted (Fig. 2).

This approach included comparing the liver transcriptome of Göttingen minipigs to those of species commonly affected by MASLD, including humans and mice. We analyzed publicly available datasets of primary human hepatocytes treated with FAs for 7 days (GSE247407), livers from C57BL/6J (B6) mice fed a high-fat diet (GSE88818), and livers of Göttingen minipigs fed a high-fat diet, to identify DEGs (Fig. 2A). Close to 700 genes were uniquely differentially expressed in Göttingen minipig livers; 90 of these remained significant after correction for multiple testing via FDR (Supplemental Table S2). To confirm the biological relevance of these differences, gene expression patterns between Göttingen minipigs and other MASLD-susceptible miniature pig breeds (Wuzhishan and Tibetan minipigs: GSE142346) fed a high-fat diet supplemented with cholesterol were compared (Fig. 2B) (12). Here, 717 genes were specific for the Göttingen minipigs, including all 692 genes identified in the first comparison, as well as 90 genes that passed FDR correction. The 692 genes with a uniquely altered expression in the liver of Göttingen minipigs were then screened for associations with disease-related pathways, overlap with candidate genes from pig GWAS, and potential regulatory TFs (Fig. 2C I-III). Finally, additional data for TF target prediction including ChIP-seq data on histone modifications - indicating active and inactive promoters and enhancers, gene-gene correlations (Fig. 2D IV-V), and network analyses (Fig. 2E) were integrated to comprehensively identify key molecular mechanisms contributing to MASLD protection.

### Transcriptomic response to high-fat diet in the liver of Göttingen minipigs

To identify protective mechanisms in Göttingen minipigs relative to species susceptible to MASLD, we performed a comparative analysis of hepatic gene expression in mice, minipigs, and primary human hepatocytes. This analysis highlighted the well-documented interspecies differences in responses to high-fat diet or exposure to FAs, revealing limited overlap in genes altered by lipid load, with only 10 genes showing consistent regulation across all three species (Fig. 2A). The divergence was even more pronounced at the pathway level, where only five biological pathways were shared among the three species, suggesting remarkably distinct adaptive responses to lipid overload. Both mouse and human liver models exhibited a shared pathway linked to PPARγ signaling and FA metabolism, consistent with enhanced lipogenesis, whereas human hepatocytes specifically displayed enrichment of pathways related to TNF signaling and alcoholic liver disease, indicating distinct processes with lipid accumulation and inflammatory responses (Supplemental Fig. S1).

**Fig. 3:**
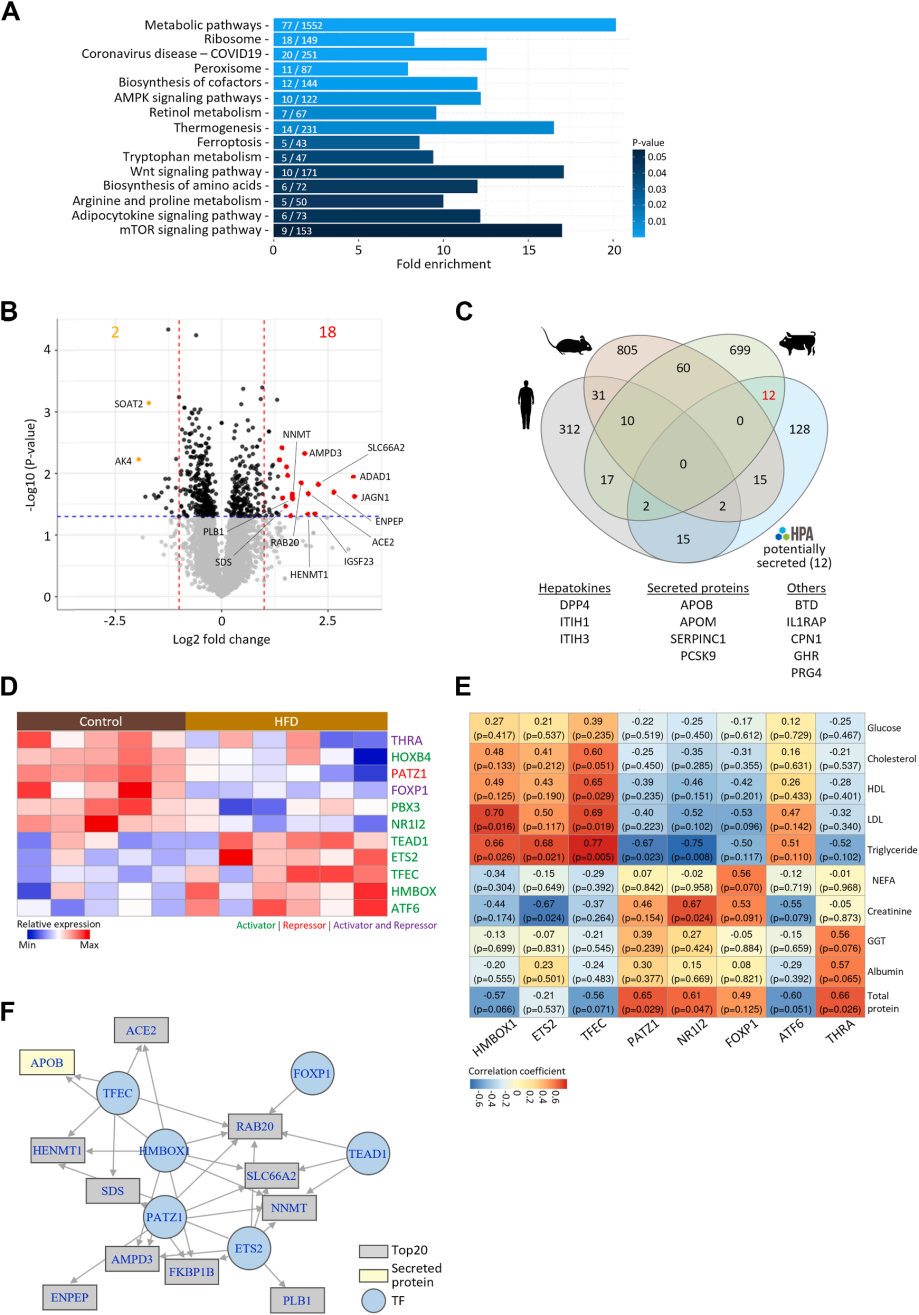
Screening for potential pathogenic signals and candidates. A: Pathway enrichment analysis of uniquely differentially expressed genes (DEGs) in Göttingen minipig livers after high-fed diet (HFD) feeding. B: Volcano plot of all DEGs in Göttingen minipig livers, highlighting unique DEGs for Göttingen minipigs with the largest fold change in response to HFD. C: Venn diagram showing overlap between 174 genes encoding predicted secreted proteins and all DEGs of livers/primary hepatocytes under HFD/high fatty acid treatment of mice, human hepatocytes, and minipigs. D: Heatmap of 11 uniquely differentially expressed transcription factors (TFs) in livers of Göttingen minipigs after HFD; TFs are color-coded by literature-based screening of activator or repressor function. E: Heatmap of Spearman correlations between TF expression in Göttingen minipig livers and selected phenotypic parameters. Colors and numbers indicate correlation coefficients, while numbers in brackets indicate corresponding *P*-values. F: Network depicting putative transcriptional regulation of transcription factors (circles) based on binding motifs in regulatory regions of the top 20 DEGs and genes coding for potential secreted proteins (rectangles – *gray*/*yellow*).

To directly pinpoint the mechanisms underlying MASLD protection in Göttingen minipigs, we analyzed pathways associated with the 692 genes uniquely differentially expressed in Göttingen minipig livers. This analysis revealed an enrichment of metabolic pathways involving retinol and amino acid metabolism, along with signaling pathways including WNT, AMPK, adipocytokine, and mTOR signaling (Fig. 3A). Together, these findings suggest that these coordinated alterations in metabolic and signaling pathways contribute to the protection against MASLD in Göttingen minipigs.

### Screening for candidates protecting against MASLD in Göttingen minipigs

To identify key molecular candidates contributing to MASLD resistance in Göttingen minipigs, we (1) prioritized genes with the highest expression changes, (2) screened for classical hepatokines and other proteins secreted by the liver with potential metabolic regulatory function, and (3) focused on TFs that may drive the observed transcriptional response. Ranking the top 20 genes by fold change revealed a predominance of upregulated genes: 18 were significantly upregulated in the livers of Göttingen minipigs following high-fat diet feeding, suggesting the activation of adaptive metabolic programs, whereas only two genes were downregulated (Fig. 3B). Specifically, the upregulated genes encompassed pathways in amino acid metabolism (*SLC66A2*, *SDS*), regulation of methylation (*NNMT*, *HENMT1*), lipid and cholesterol metabolism (*PLB1*, *SOAT2*), transcriptional regulation (*ZBED6*, *ENSSSCG00005027610*), and immune response (*JAGN1*, *Igkv15-103*; Supplemental Table S3). Notably, *SOAT2* was among the downregulated genes; the knockout of this gene in mice promotes lipid oxidation and decreases hepatic fat storage (22), consistent with the MASLD-resistant phenotype of Göttingen minipigs.

To identify affected secreted proteins, which potentially exert systemic metabolic effects, we utilized the Human Protein Atlas (23, 24) and compiled a list of 174 genes enriched in livers. Of these 174 genes encoding secreted proteins, 12 were differentially expressed exclusively in Göttingen minipig liver (Fig. 3C), including three genes that remained significant after correction for multiple testing (*APOM*, *BTD,* and *PRG4*). Among these, three encode classical hepatokines (DPP4, ITIH1, and ITIH3), all of which were expressed at lower levels in Göttingen minipigs (Supplemental Fig. S2). Notably, while decreased expression of *DPP4* may contribute to protective mechanisms (25), the reduction in *ITIH3* expression was unexpected, as prior studies suggest that higher *ITIH3* expression decreases de novo lipogenesis and mitochondrial bioenergetics (26). This discrepancy warrants further investigation to clarify the role of ITIH3 in the Göttingen minipig liver and to reconcile these findings with existing knowledge.

Other candidates, which are liver-derived plasma proteins involved in lipid transport, coagulation, and cholesterol metabolism, respectively, include APOB, APOM, SERPINC1, and PCSK9 (27, 28, 29), indicating a potential role in mediating systemic metabolic effects. Notably, PCSK9, which promotes LDL-receptor degradation, was strongly downregulated in response to high-fat diet (Supplemental Fig. S2). Beyond its established plasma LDL-lowering effects, reduced PCSK9 activity has also been reported to protect against MASLD by limiting hepatic lipid accumulation (30).

Furthermore, we identified a set of five DEGs predicted to encode secreted proteins, including *CPN*, the growth hormone receptor, and *PRG4*, a proteoglycan that has not previously been reported to be secreted, it was predicted to possess secretory properties. The remaining two genes (*BTD*, *IL1RAP*) encode proteins known to be secreted and are involved in biotin recycling and modulation of interleukin-1 signaling, respectively (31, 32).

Finally, 11 TFs were identified as uniquely differentially expressed in the livers of Göttingen minipigs in response to a high-fat diet. These include five upregulated activators (*TEAD1*, *ETS2*, *TFEC*, *HMBOX1*, and *ATF6*) and six downregulated TFs, comprising three activators (*PBX3*, *HOXB4*, and *NR1I2*), one repressor (*PATZ1*), and two with both repressor and activator functions (*FOXP1* and *THRA*) (Fig. 3D). *TFEC* expression was validated by qRT-PCR (Supplemental Fig. S3), and the biological relevance of the remaining TFs was further supported by correlation with MASLD-associated phenotypes (Fig. 3E). Interestingly, several TFs identified through the cross-species transcriptomic integration approach showed coherent associations with metabolic and liver-related traits. In particular, *ETS2*, *ATF6*, *TFEC*, and *HMBOX1* were predominantly positively correlated with lipid-associated parameters, including triglycerides, LDL, HDL, and cholesterol levels, while simultaneously showing negative correlations with creatinine and total protein levels. Among these, *TFEC* transcript levels exhibited particularly strong positive correlations with triglycerides (r = 0.77), LDL (r = 0.69), HDL (r = 0.65), and cholesterol (r = 0.60) (Fig. 3E).

In contrast, *THRA*, *FOXP1*, *NR1I2*, and *PATZ1* transcript levels exhibited an inverse correlation pattern, characterized by negative associations with lipid-related traits and positive associations with parameters such as creatinine, total protein, and albumin. For example, *NR1I2* demonstrated strong negative correlation with triglycerides (r = −0.75) and positive correlations with creatinine (r = 0.67) and total protein (r = 0.61).

Notably, the observed correlations were not randomly distributed but instead revealed distinct TF clusters with coherent phenotype association patterns. These findings provide additional evidence supporting the biological relevance of the identified TFs in MASLD-associated alterations, despite the limited statistical power inherent to low-sample-size transcriptomic datasets.

Taken together, these findings, the downregulation of *SOAT2*, the altered expression of genes encoding hepatokines such as *DPP4* and secreted proteins (*PCSK9*, *APOB*), and the involvement of multiple TFs, highlight the complexity of the molecular mechanisms underlying MASLD resistance in Göttingen minipigs and underscore the value of identifying species-specific pathways.

### A complex gene regulatory network is linked to MASLD protection

To evaluate the putative roles of the identified TFs in MASLD protection, we applied computational approaches to predict which DEGs are directly regulated by these TFs. This analysis integrated information on accessible DNA regions (open chromatin regions), correlations between DEG and TF expression patterns, and the presence of specific DNA binding motifs. Collectively, 11 TFs were predicted to influence the expression of 477 DEGs (Fig. 2D). Notably, the analysis not only identified TF-specific target genes but also revealed a core regulatory network composed of TFEC, HMBOX1, PATZ1, ETS2, TEAD1, and FOXP1, predicted to regulate at least 10 of the top 20 upregulated genes and the secreted protein APOB (Fig. 3F). Targets include AMPD3, which contributes to cellular energy homeostasis by modulating ATP/ADP and ATP/AMP ratios, thereby potentially activating AMPK (33). Furthermore, the TF NR1I2 was predicted to repress both *SOAT2*, previously linked to protection against MASLD, and *ITIH3*, along with several other genes encoding secreted proteins, including *APOM*, *SERPINC1*, and *ITIH1* (Supplemental Fig. S4A).

To connect these transcriptional changes to observable phenotypes, we queried the pig GWAS biobank created by Zeng *et al.* (6) for associations between the candidate TFs and genes with various MASLD-related phenotypes. The pig GWAS biobank comprises 231 traits based on 62,961 pigs representing more than 100 breeds. Here, we focused on liver-related traits, including cholesterol metabolism, FA levels, and general liver health parameters (Supplemental Fig. S5). Among the top 20 candidates, significant associations were identified for five TFs, six upregulated genes, two downregulated genes, and one gene encoding the secreted protein APOB. Genetic variants in *APOB* were associated with altered LDL-cholesterol levels, consistent with its established role in LDL transport (34).

Overall, across the top 20 genes, the Human Protein Atlas-derived secreted-protein set, and the TF set, a notable proportion were linked to phenotypes indicative of liver protection or improved hepatic metabolic health.

### The TF ETS2 may mediate effects of MASLD resistance via AMPK signaling

**Fig. 4:**
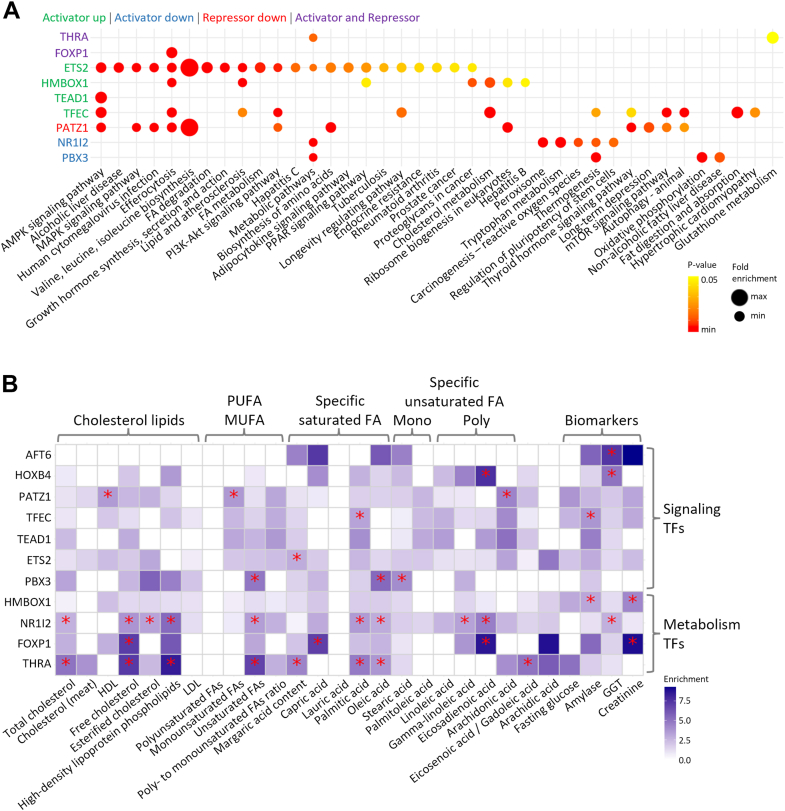
Pathway enrichment analysis of transcription factor targets and their association with specific traits. A: Pathway enrichment analysis of putative target genes of nine transcription factors (TFs) uniquely differently expressed in Göttingen minipig liver. B: Enrichment of TF targets among pig GWAS for lipid classes and biomarkers. The color scale depicts enrichment values; asterisks denote significant enrichment (*P*-value < 0.05). GWAS, genome-wide association study.

In order to identify further processes contributing to protection against MASLD, we performed pathway enrichment analysis of the target gene sets (Fig. 4A). Among the 11 TFs, targets of nine TFs were significantly enriched for multiple signaling pathways, whereas targets of ATF6 and HOXB4 showed no significant enrichment. Interestingly, several genes (e.g., *RHEB* and *STRAD*) involved in AMPK signaling were predicted to be regulated by subsets of TFs such as ETS2, TEAD1, TFEC, and PATZ1. Notably, ETS2 emerged as key regulator, as it was predicted to control six of the seven genes associated with the AMPK signaling pathway (*ADIPOR2*, *CPT1A*, *IGF1*, *RAB14*, *RHEB*, *TBC1D1*; Supplemental Fig. S6). Consistent with this, *AMPD3*, previously identified among the strongly altered genes and known to modulate AMPK signaling, was also differentially expressed, further supporting a central role for this pathway in MASLD resistance. In addition, the TFEC and PATZ1 exhibited shared targets in pathways related to thyroid hormone and mTOR signaling, suggesting a primary role in signal transduction rather than metabolism (Fig. 4A). In contrast, THRA, NR1I2, and PBX3 showed enrichment of targets directly linked to metabolic processes, including tryptophan and glutathione metabolism (Fig. 4A).

A similar pattern was observed when examining enrichment of TF targets for pig GWAS traits related to cholesterol and lipid metabolism (Fig. 4B). Here, NR1I2, FOXP1, and THRA appeared to play significant roles in regulating genes involved in FA or cholesterol metabolism, while other TFs showed comparatively weaker associations with these specific lipid classes. This supports a well-established biological concept that activation of specific TFs can drive alterations in signaling or metabolic pathways through regulation of their downstream target genes (35).

Collectively, these findings highlight a central role of AMPK signaling in conferring protection against MASLD, with ETS2 emerging as a candidate regulator of this pathway. They also delineate diverse roles for other TFs, such as TFEC and PATZ1 in signaling transduction, and NR1I2, FOXP1, and THRA in metabolic processes.

### Network analysis detects a set of highly relevant gene modules

**Fig. 5:**
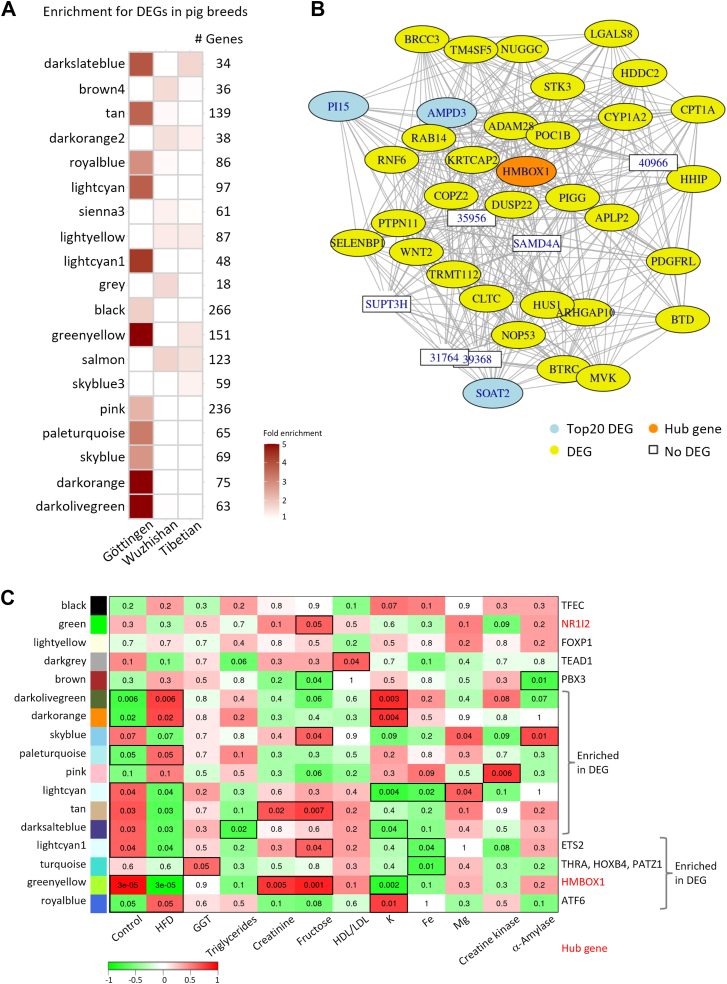
WGCNA of genes expressed in livers of Göttingen minipigs. A: Heatmap depicting fold enrichment in WGCNA modules for differentially expressed genes (DEGs) in livers of Göttingen minipigs fed a high-fat diet (HFD) and livers of Tibetian and Wuzhishan minipigs fed a HFD supplemented with cholesterol. B: Coexpression network of the *greenyellow module*. Node color indicates DEGs (*yellow*), top 20 DEGs (*lightblue*), and hub genes (*orange*). C: Heatmap of correlations between module eigengenes and MASLD-relevant phenotypic traits. Numbers indicate corresponding *P*-values; the color scale represents correlation coefficients. Significant correlations are indicated by *black labels*. WGCNA, weighted gene coexpression network analysis.

To identify groups of genes with coordinated expression patterns in the livers of Göttingen minipigs that may contribute to their protective phenotype, we performed WGCNA. This analysis identified 50 modules, each comprising between 34 and 2,665 genes. Among these, 19 modules were significantly enriched for DEGs identified in Göttingen minipigs or other minipig breeds (Fig. 5A).

Two hub genes, *HMBOX1* (greenyellow) and *NR1I2* (green), were previously identified as key TFs that were positively and negatively associated with lipid-related parameters, respectively. The top 40 genes ranked by module membership within the greenyellow module are shown in Fig. 5B, supporting a central regulatory role of *HMBOX1* within this module. Notably, several genes connected to *HMBOX1*, including *PI15*, *AMPD3*, and *SOAT2*, were also among the top 20 genes exhibiting the largest fold changes.

Overall, the greenyellow, darkorange, and darkolivegreen modules were highly enriched for DEGs specific to Göttingen minipigs, suggesting that these modules may contribute substantially to the protective liver phenotype (Fig. 5A). Genes within these modules were predominantly associated with FA metabolism, carbon and pyruvate metabolism, and lipid metabolic processes, indicating a primary metabolic rather than inflammatory or signaling-related functions (Supplemental Table S5).

In contrast, modules enriched for DEGs in the two MASLD-susceptible minipig breeds (darkorange2, lightyellow, and salmon) were associated with pathways involved in cytokine-mediated signaling, protein transport, and immune system responses, suggesting potential contributions to MASLD progression.

To integrate the identified TFs into broader transcriptional programs, we examined correlations between module eigengenes and TF expression levels (Fig. 5C). The greenyellow and green modules displayed opposing association patterns that mirrored the phenotype correlations previously observed for their corresponding hub TFs. Specifically, *HMBOX1*, identified as a hub gene within the greenyellow module, clustered with *ETS2*, *ATF6*, and *TFEC*, all of which showed predominantly positive associations with lipid-related phenotypes. In contrast, the green module, centered on the hub TF *NR1I2*, clustered with *THRA*, *PATZ1*, *HOXB4*, and *FOXP1*, which displayed inverse associations with lipid-related parameters.

To assess the conservation of coexpression structures between Göttingen minipigs and MASLD-susceptible minipig breeds, we performed module preservation analysis (Supplemental Fig. S7). While several modules showed moderate preservation across breeds, the overall preservation statistics (D = 0.63–0.65) indicated substantial differences in network organization. Notably, modules enriched in Göttingen minipig-specific DEGs displayed distinct correlation structures compared to susceptible breeds, suggesting selective transcriptional rewiring rather than complete loss of conserved metabolic programs. Collectively, these findings further support the hypothesis that Göttingen minipigs adapt to high-fat diet conditions primarily through coordinated metabolic network remodeling.

Together, these results indicate that the identified modules represent coordinated transcriptional programs linked to distinct metabolic states. Importantly, modules enriched in Göttingen minipig-specific DEGs were primarily associated with metabolic pathways rather than inflammatory signaling, supporting the hypothesis that metabolic adaptation and transcriptional rewiring contribute to MASLD resistance in Göttingen minipigs.

### Transcriptional impact of differentially expressed TFs within modules

**Fig. 6:**
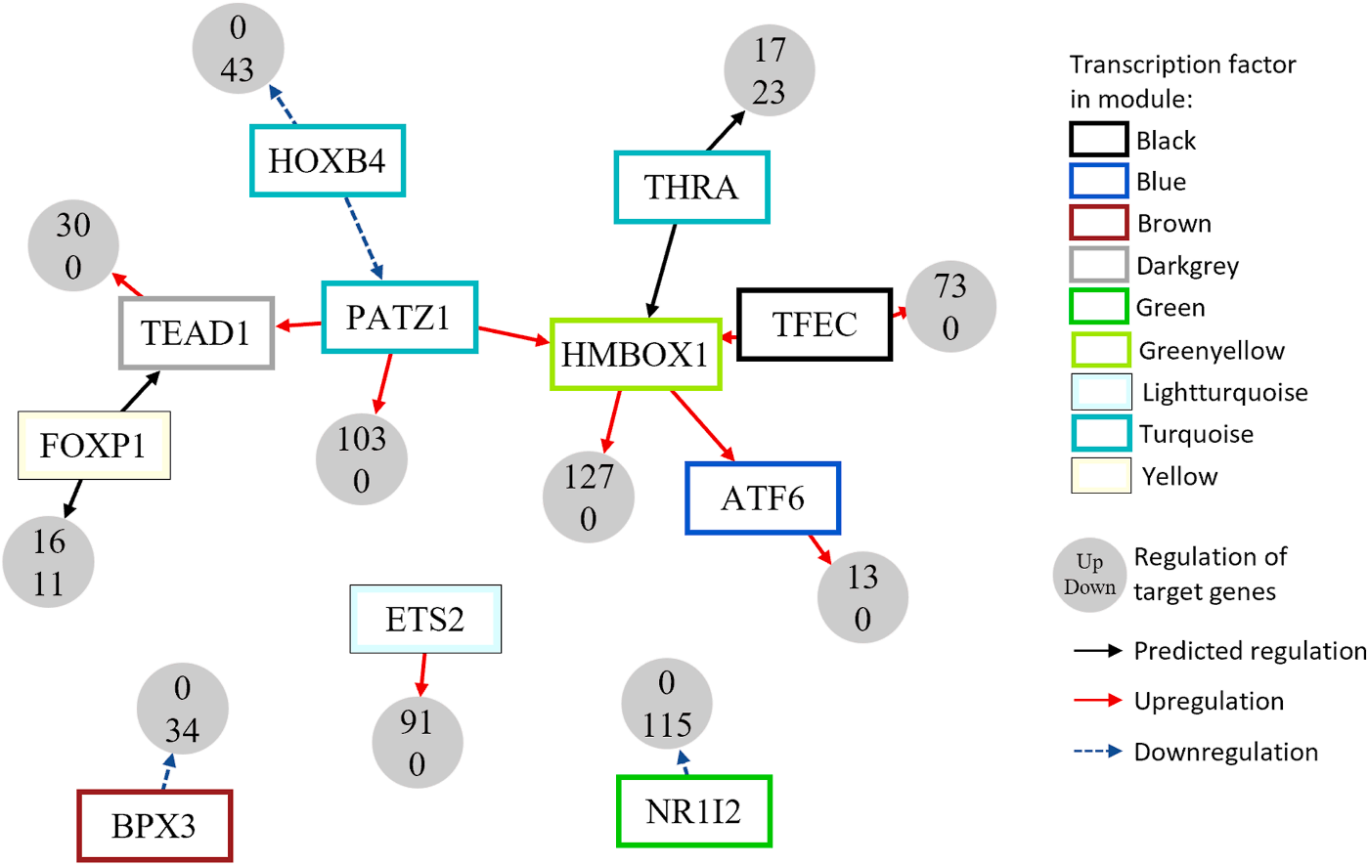
Coexpression networks with corresponding transcription factors and numbers of potential targets. Rectangles represent transcription factors; frame color indicates module membership. Circles indicate the number of regulated genes. *Red solid lines* denote upregulation of target genes following high-fat diet feeding, *blue dashed lines* represent downregulation, and *black solid lines* indicate interactions with unclear effects on gene expression. A color key explains module assignments.

To further access the overall landscape of transcriptional regulation and establish a hierarchical structure of the identified coexpression networks, we examined the distribution of the 11 previously identified TFs across the WGCNA modules. Eight TFs were interconnected within six modules (turquoise, darkgrey, yellow, green, greenyellow, black, and blue), together comprising 284 genes that may be regulated by these TFs (Fig. 6). For example, *PATZ1* of the turquoise module may upregulate *HMBOX1* within the greenyellow module, which in turn affects *ATF6* in the blue module. This hierarchical organization showed partial similarity to the network of regulators for highly relevant genes (Fig. 3E). Moreover, the central TFs *HMBOX1* and *PATZ1* were predicted to regulate other TFs (TFEC/ATF6 and TEAD1/HMBOX1) and together were associated with the regulation of more than 100 target genes, highlighting their potential roles as key upstream regulators within the transcriptional network.

To validate the identified central TFs, we utilized the GEOdataset GS5215711, which reports ChIPsSeq of PATZ1 in HepG2 cells. In general, this dataset revealed PATZ1 binding sites within the promoter region of 80 out of 103 predicted PATZ1 target genes (Supplemental Table S4). Interestingly, *HMBOX1* was among these targets, whereas no PATZ1 binding site was detected in the promoter region of *AMPD3*. In addition, *HMBOX1* was classified as the hub gene of the greenyellow module, which showed the strongest enrichment for DEGs, further highlighting its role in MASLD protection. In contrast, the three modules (brown, light turquoise, and green) associated with the remaining three TFs (PBX3, ETS2, and NR1I2) showed no interconnectivity. However, *NR1I2*, the hub gene of the green module, was predicted to target 115 genes involved in metabolic pathways, suggesting a potential contribution to MASLD resistance (Fig. 6; Supplemental Table S5).

To assess whether the identified TF targets were nonrandomly integrated into the detected coexpression modules, we compared mean module membership values of predicted TF targets to 1,000 permutations of randomly selected target sets. Targets of several TFs, particularly *HMBOX1* and *ETS2*, displayed higher average module membership values compared to random expectations, supporting their integration into coherent transcriptional programs rather than representing randomly distributed target genes (Supplemental Table S6). These findings further strengthen the proposed central regulatory role of *HMBOX1* and *ETS2* within the protective metabolic network of Göttingen minipigs.

Overall, the network shown in Figure 6 supports an important regulatory role of PATZ1 and HMBOX1 in inhibiting the development of MASLD in Göttingen minipigs, as approximately 41% (284 genes) of the uniquely affected genes in Göttingen minipig liver appear to be regulated by these two proteins.

## Discussion

The applied multispecies approach—combining candidate screening based on fold change (top 20), assessment of affected secreted proteins, unbiased coexpression network analysis, and TF target prediction—independently converged on a set of regulatory networks potentially associated with liver-protective mechanisms of Göttingen minipigs. These networks involve key TFs ETS2, HMBOX1/PATZ1, and NR1I2, which mediate downstream regulatory effects linked to AMPK signaling, lipid metabolism (Fig. 4A), and the liver secretome (Fig. 3C), respectively. Importantly, integrating phenotype correlations with coexpression module analyses revealed coherent transcriptional programs associated with distinct metabolic states, further supporting the biological relevance of the identified TFs despite the limited statistical power inherent to low-sample-size transcriptomic datasets. Moreover, the incorporation of ChIP-seq data to validate TF-target predictions strengthens confidence in the inferred interactions.

The findings notably support a potential involvement of AMPK signaling pathway in conferring protection against MASLD. ETS2 emerged as a candidate regulator linked to this pathway (Fig. 4A; Supplemental Fig. S6), consistent with prior studies establishing AMPK as a critical regulator of cellular energy metabolism and a promising therapeutic target for MASLD (36, 37). Importantly, the transcriptional regulation of AMPK signaling via ETS2 has not previously been reported. While ETS2 is primarily characterized by its role in macrophage-mediated inflammation or its suppression (38, 39), it was also expressed in the primary hepatocytes used in our study. We therefore propose that ETS2 mediates effects on AMPK signaling through its predicted targets, a hypothesis warranting further experimental validation. Furthermore, the upregulation of *AMPD3*, a known modulator of AMPK activity (33), in livers of Göttingen minipigs, and its predicted transcriptional regulation by HMBOX1 and PATZ1 (Fig. 3B, E), provides additional support for the interconnected nature of these regulatory networks.

The identification of HMBOX1 and PATZ1 as key transcriptional regulators in MASLD-protective networks is particularly compelling. These TFs were predicted to regulate a broad set of target genes, including additional TFs such as ATF6 and TFEC (Fig. 6), indicating a complex, hierarchical regulatory network that may contribute to liver homeostasis and protect against hepatic lipid accumulation. HMBOX1, ETS2, ATF6, and TFEC clustered together in the module eigengene analyses and showed predominantly positive associations with lipid-related phenotypes, whereas NR1I2, THRA, PATZ1, and FOXP1 formed an opposing cluster characterized by inverse associations with lipid-related parameters. These findings suggest that the identified TFs participate in coordinated transcriptional programs rather than isolated signaling events. Notably, HMBOX and PATZ1 have previously been described as inhibitors of liver cancer and tumor suppressors, respectively (40, 41), suggesting a broader protective role beyond metabolic regulation. The fact that both TFs are located in distinct WGCNA modules enriched in DEGs (Fig. 5A) further supports their potential central roles within the identified protective transcriptional networks.

In addition, the TF NR1I2 (also known as pregnane X receptor, PXR), emerged as a hub gene in the green WGCNA module, which was enriched for genes related to metabolic pathways (Supplemental Table S5). Among the 115 genes predicted to be upregulated by *NR1I2* were several coding for liver-secreted proteins, including *SERPINC1*, *ITIH1/3*, and *APOM* (Fig. 3C), as well as *SOAT2*, which, when deleted in mice, has been shown to confer protection against lipid accumulation (22). This aligns with the established role of nuclear receptors, such as *THRB* and *HNF4A*, in modulating lipid and glucose metabolism (35, 42) and suggests that *NR1I2* is a promising candidate for in-depth exploration. More broadly, the observed module preservation and eigengene analyses suggest that Göttingen minipigs do not simply lack pathogenic pathways seen in susceptible breeds, but rather exhibit selective metabolic transcriptional rewiring of metabolic programs characterized by coordinated adaptation of regulatory and metabolic networks.

Furthermore, the reduced mRNA expression levels of the secreted proteins PCSK9 and DPP4 in MASLD-resistant Göttingen minipigs after high-fat diet feeding may also contribute to protection from hepatic lipid accumulation. Hepatic overexpression of *DPP4* in mice induced fatty liver (43) and inhibition of *PCSK9* has been shown to decrease hepatic fat content (30). These observations suggest that, in addition to key TFs, specific secreted proteins may also contribute to suppression of MASLD development.

In conclusion, our study provides novel insights into mechanisms of MASLD protection by focusing on transcriptomic adaptations in the livers of resistant animal models. By integrating cross-species transcriptomics, pathway enrichment analyses, phenotype correlations, TF-target prediction, and coexpression network analyses, we identified coordinated regulatory networks that may underlie the resistance of Göttingen minipigs to hepatic lipid accumulation. Overall, our findings highlight the value of integrative multispecies systems biology approaches for identifying candidate pathways and regulatory networks involved in MASLD resistance and provide a resource for future mechanistic and translational studies aimed at preventing MASLD development.

### Limitations

The identification of NNMT and HENMT1, both associated with DNA and RNA methylation (44, 45), among the top 20 DEGs underscores the need for a more comprehensive systems-level approach. Our current focus on transcriptomic data represents a key limitation. Future investigations incorporating epigenomic (e.g., DNA methylation) together with proteomic datasets, will be essential to more thoroughly elucidate these complex regulatory mechanisms and to rigorously validate predicted TF-target interactions.

## Data availability statement

All RNA sequencing data described in this article can be found on GEO under accession numbers: GSE142346, GSE88818, and GSE247407. Details regarding data processing and sample information are provided in the Methods section.

## Supplemental data

This article contains supplemental data.

## Conflict of interest

The authors declare that they have no conflicts of interest with the contents of this article.
