# Supplementary material for: Multispecies transcriptome analysis identifies networks protecting against MASLD in Göttingen minipigs
Source: J Lipid Res. 2026 Jun 25;67(8):101089. doi: 10.1016/j.jlr.2026.101089 (PMC13453441; doi:10.1016/j.jlr.2026.101089)
Supplement: Supplementary file 1 [file mmc1.pdf]

Supplemental material for

Multispecies transcriptome analysis identifies networks protecting against MASLD in Göttingen minipigs

Pascal Gottmann, Wenke Jonas, Simone Renner, Julia Philippou-Massier, Theresa Hommel, Maik Dahlhoff, Anja Zeigerer, Eckhard Wolf, Heike Vogel, Annette Schürmann

Corresponding author: Pascal Gottmann (Passi.g@web.de)

Supplemental Table

Supplementary Table S1. RT-qPCR Primer

	**Forward 5'- 3'**	**Reverse 5'- 3'**
ETS2	GCTGACCCAGATGAGGTGGC	CAGCAGGTTCTGGAGGTCGC
HOXB4	CGACCCCAAGTTCCCTCCGT	TGGAAGCTGCTCTCTCGCCT
HMBOX1	ACCTCCCCACAGCCTTGTACT	TCACACTGCTGTCCCTCCTCA
PATZ1	GGACCTATGGCAACAAAGAAGGC	GGTCTTCAGGTCGCTGGCAT
TFEC	AAGACTCACTGACCCAGTACAGA	ATTGTCCCCTCAGTGGTGGT
NR1I2	GACGTTACCACCAAGCAGTCCA	TGTCCCCACATACACGGCAG
FOXP1	ACGTGCCCATTTCTTCAGCA	TGGAGATTCGAGAATGGCCTGC
TEAD1	GCCGCTGCTTCATTGCACATT	AAGCCGAAGCCAAAGGGAAGT
ATF6	AGAATGGATCACTGAAGCGGCA	TGAGGGGACAGCACCTCAT
THRA	CCATGCCGGACGGAGACAAG	GTCTTCGCAAGGCAGCTCGG
B2M	CAAGATAGTTAAGTGGGATCG	TGGTAACATCAATACGATTTC
ACTB	GGATGCAGAAGGAGATCACG	ATCTGCTGGAAGGTGGACAG

Supplemental Figures

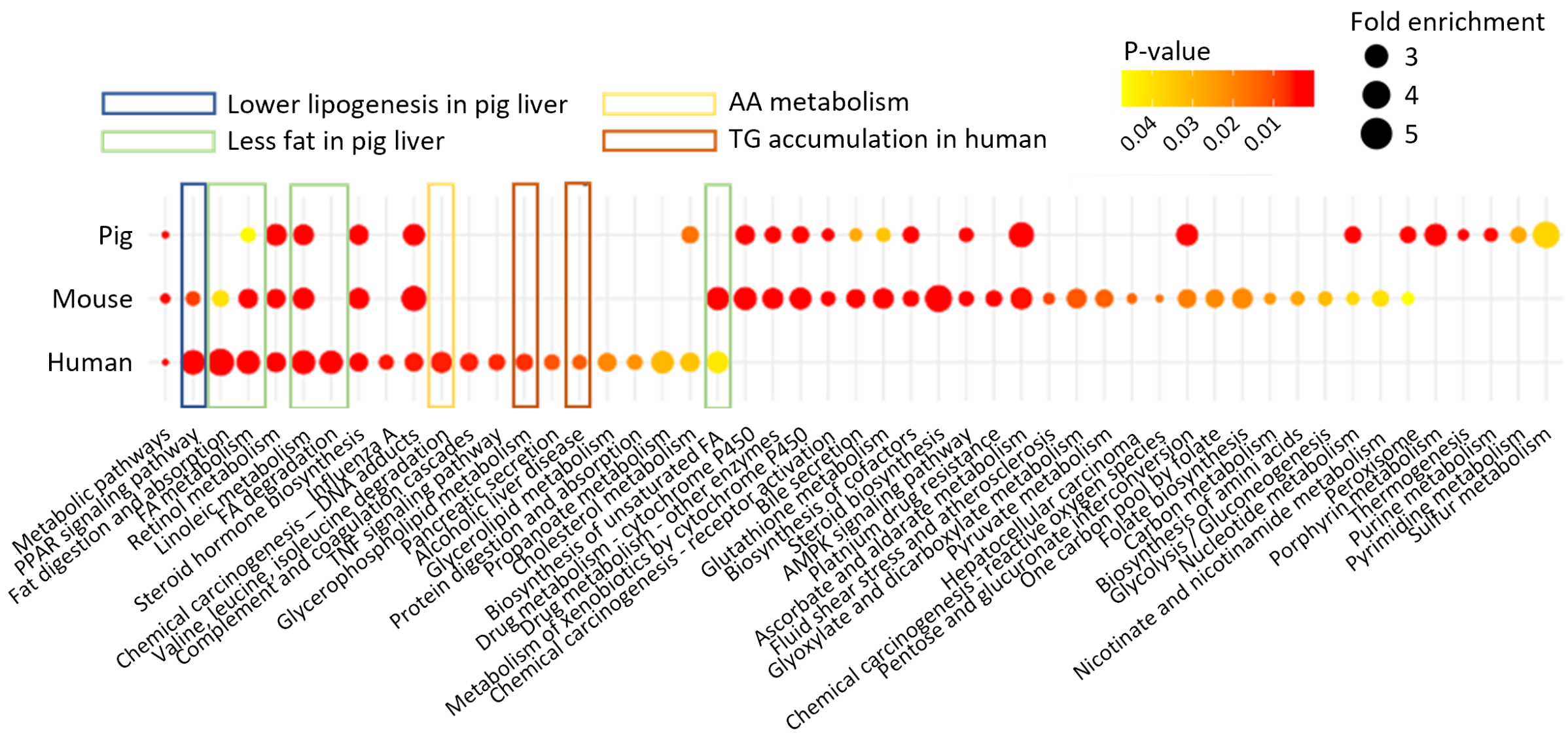

Supplemental Figure S1. Dot plot depicting enrichment of differentially expressed genes in livers or primary hepatocytes of different species after high-fat diet feeding or fatty acid treatment. The size of the dots reflects the fold enrichment, and color code reflects the p-value. Interesting pathways are marked by colored rectangles.

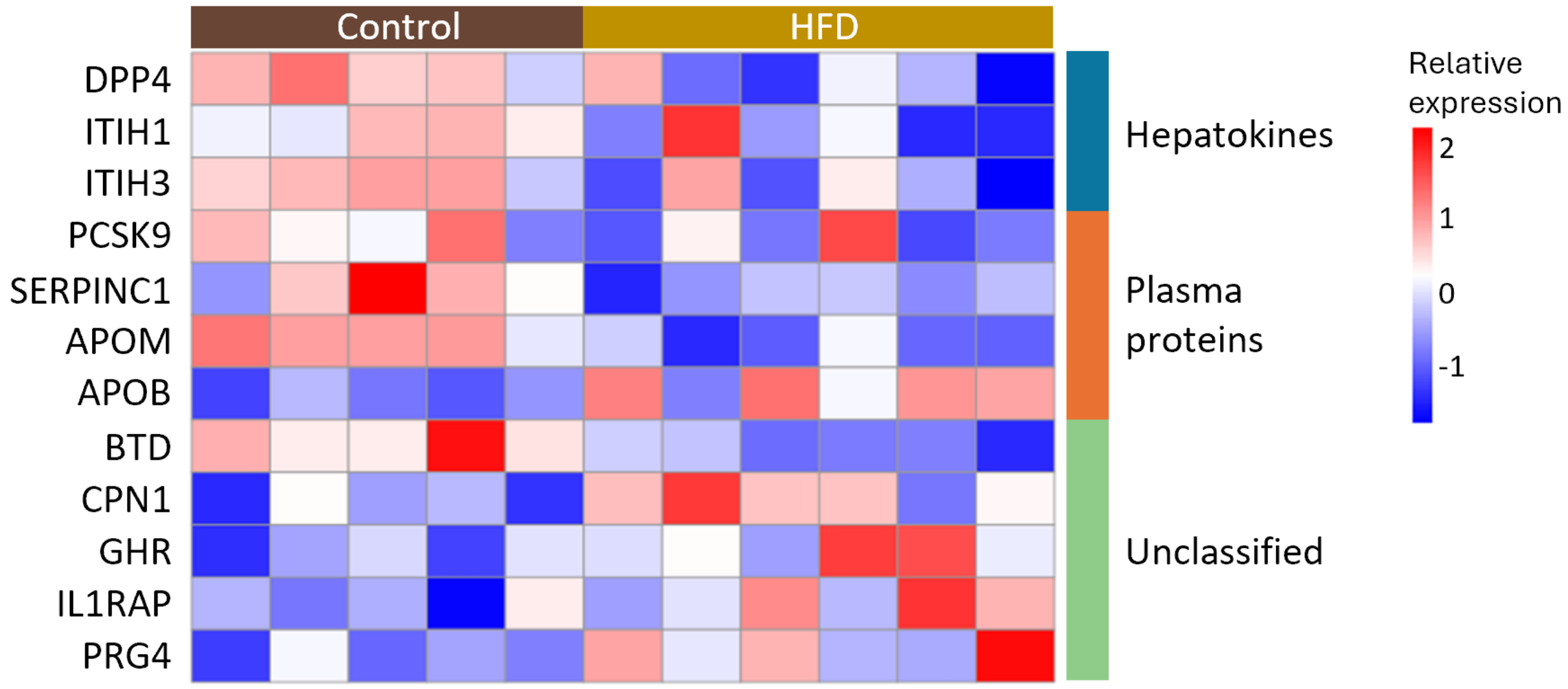

Supplemental Figure S2. Heatmap depicting changes in the expression of genes predicted to encode secreted proteins in response to a high-fat diet in the livers of Göttingen minipigs. Blue indicates known hepatokines; orange, genes encoding known plasma proteins; and green, genes for which no clear classification has been described.

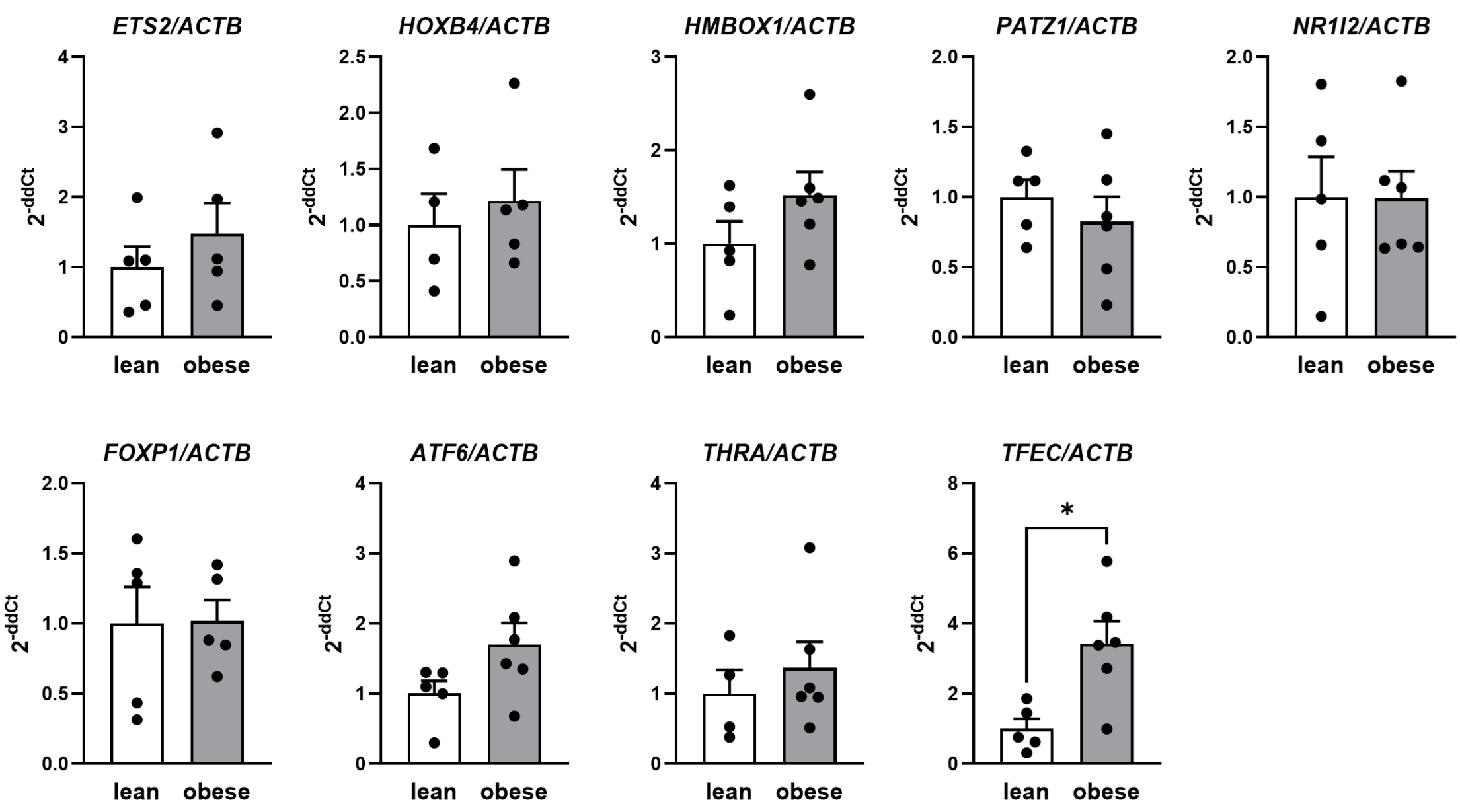

Supplemental Figure S3. Expression analysis of selected transcription factors measured by RT-qPCR in livers of obese and lean MASLD-resistant Göttingen minipigs (n = 5–6 per group; $p < 0.05$).

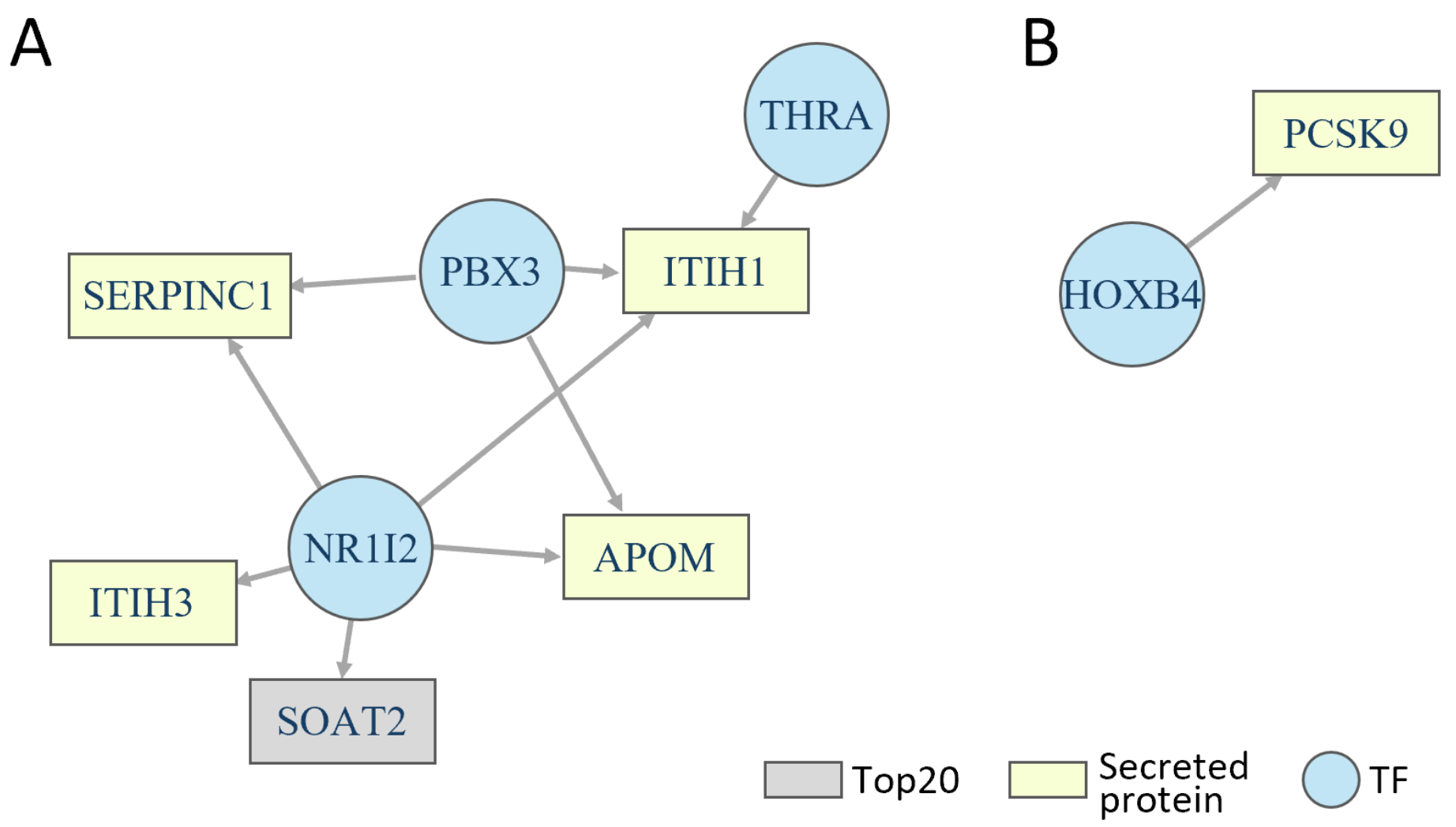

Supplemental Figure S4. Networks (A and B) indicating the predicted transcriptional regulation of key transcription factors.

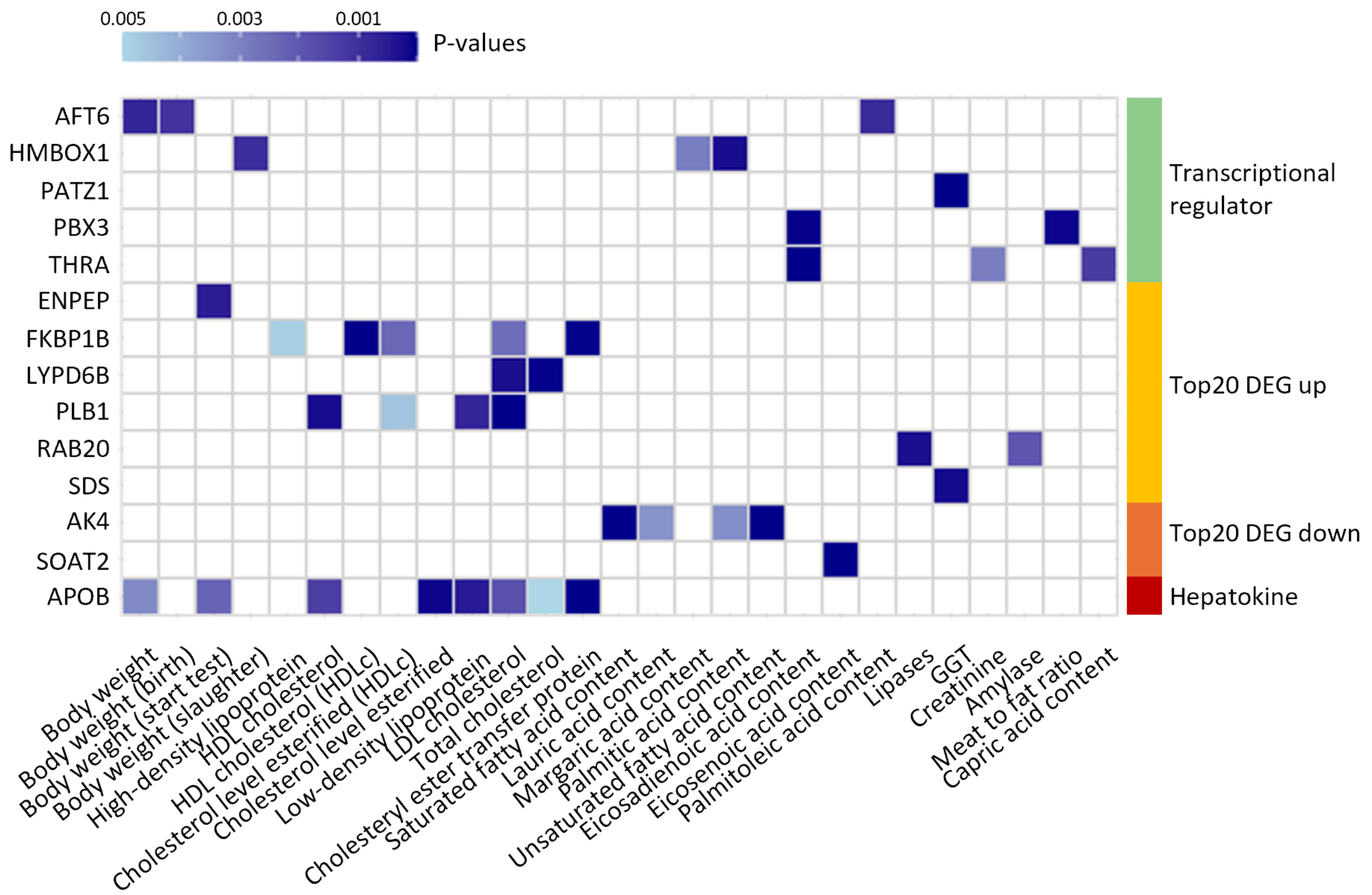

Supplemental Figure S5. Heatmap showing the associations of the candidate genes for protecting against MASLD across phenotypes in the pig GWAS.

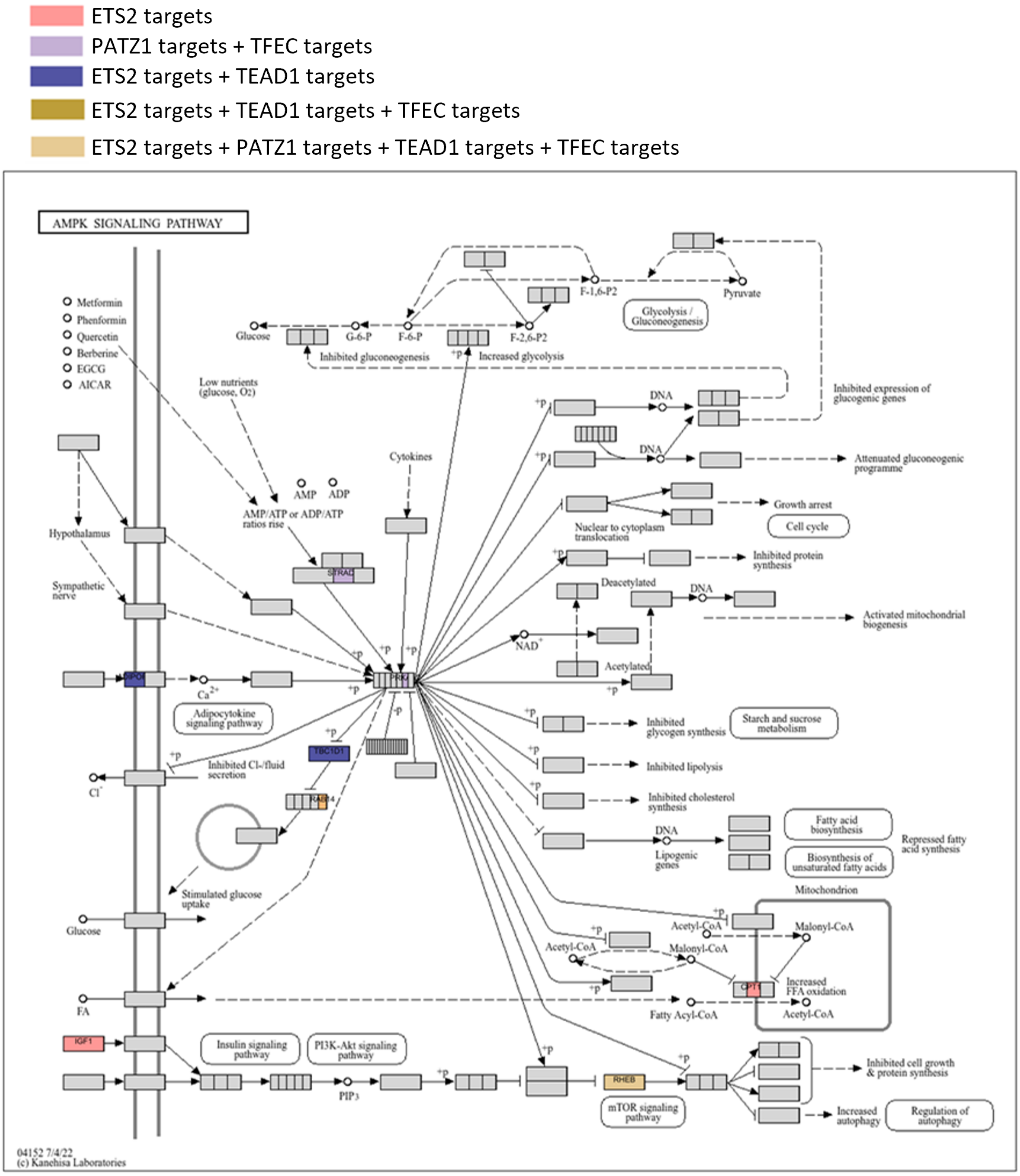

Supplemental Figure S6. AMPK-signaling pathway derived from KEGG using the Meta-KEGG tool. Color code indicates transcription factor target genes.

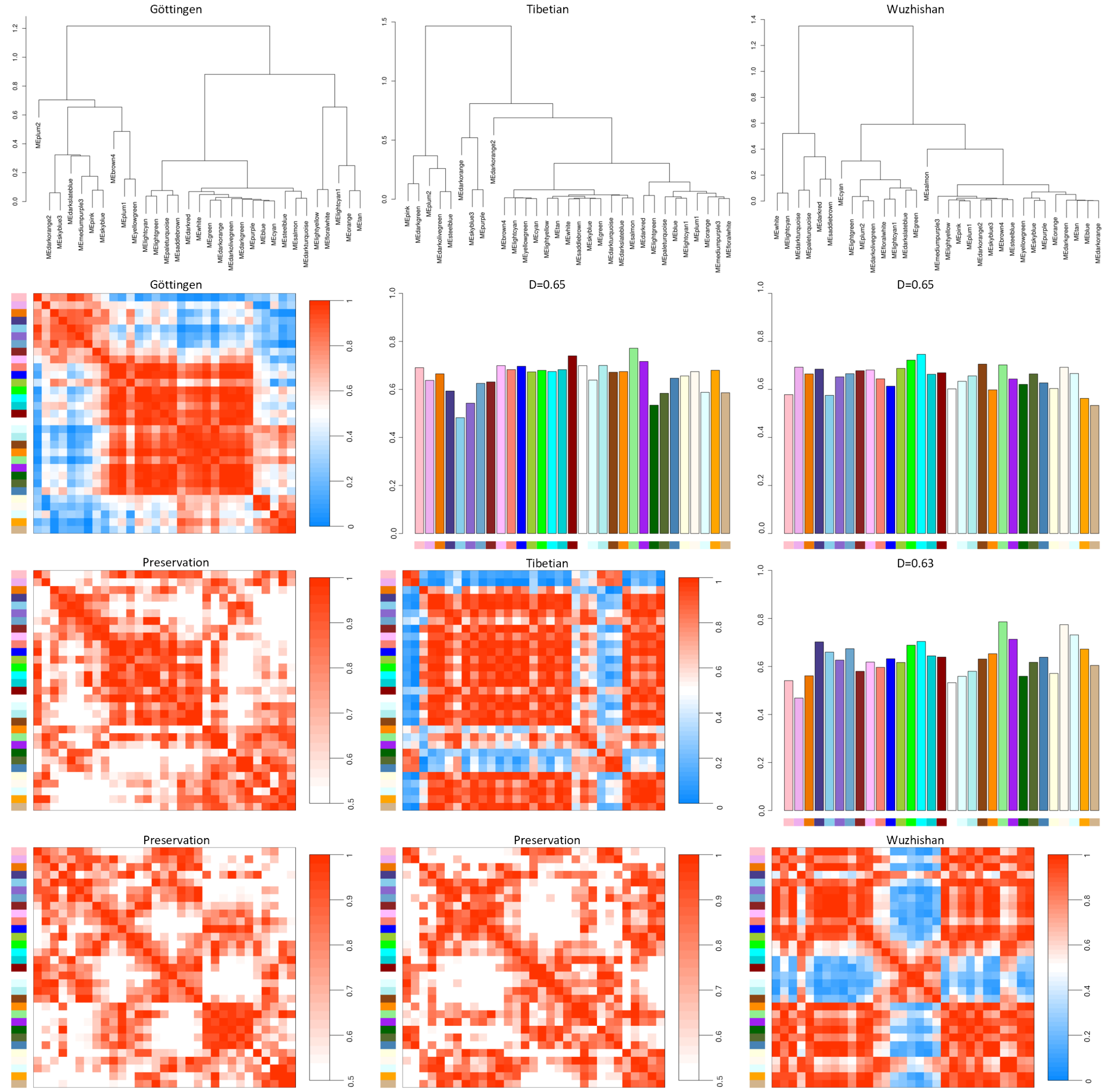

Supplemental Figure S7. WGCNA module preservation analysis across Göttingen, Tibetan, and Wuzhishan minipigs. The upper panels depict the hierarchical clustering of module eigengenes, and the lower panels display the eigengene correlation matrices and the module preservation statistics between breeds.
